# Effects of Foot-Toe Orthoses on Moment and Range of Motion of Knee Joint in Individuals with Hallux Valgus

**DOI:** 10.3390/life13051162

**Published:** 2023-05-11

**Authors:** Yongwook Kim

**Affiliations:** Department of Physical Therapy, College of Medical Sciences, Jeonju University, 303 Cheonjam-ro, Wansan-gu, Jeonju 55069, Republic of Korea; ptkim@jj.ac.kr

**Keywords:** corrective orthosis, hallux valgus, knee joint, moments

## Abstract

Although various types of hallux valgus (HV) orthoses have been used to manage hallux valgus deformity, few previous studies have determined the biomechanical effects of applying a foot-toe orthosis as a therapeutic intervention for HV deformity on the kinetics and kinematics of the knee joint. Biomechanical variables were collected from 24 patients with HV. A three-dimensional motion capture system and force platforms were used to analyze the kinetic and kinematic variables in HV orthosis conditions during gait. To determine the biomechanical effect of each orthosis for HV on knee kinetic and kinematic values, repeated-measures ANOVA was used. The knee adduction moment was significantly decreased under a hard plastic orthosis (HPO) condition compared to that under a without foot-toe orthosis (WTO) condition (*p* = 0.004). There was a significant decrease in maximal external rotation of the knee joint in HPO than in WTO at the stance phase during gait (*p* = 0.021). All of the kinetic and kinematic data showed no significant differences between WTO and soft silicone orthosis conditions (*p* > 0.05). This study indicates that a stronger foot-toe orthosis, such as HPO, to correct HV deformity has a positive effect on the moment and joint motion occurring in the knee joint during walking. In particular, the application of this type of HV orthosis can reduce knee adduction moments associated with the development and progression of knee OA.

## 1. Introduction

Hallux valgus (HV) is a toe–foot deformation identified by the first metatarsophalangeal (MTP) joint deviating inside and the hallux protruding laterally towards the second toe [1,2]. Although composite factors related to the pathogenesis of HV deformity are known [3,4], the exact cause of HV development has not been clarified yet. The etiopathogenesis of HV is considered to include malalignment and hypermobility of the first intermetatarsal and MTP joints, and the incidence is high not only in the general population but also in ballet dancers [1,2]. Knee osteoarthritis (OA) is a musculoskeletal disorder of the lower extremities that can cause knee joint pain and dysfunction [5]. Local knee malalignment is a mechanical factor affecting the progression of knee OA [6]. Deformed toe–foot postures, including HV, pes planus, and pronated rearfoot, are more frequently found in patients with knee OA than those in a control group [5,7,8]. An abnormal HV angle is associated with the presence of patellofemoral pain syndrome [9], and medial knee OA has been associated with same-side HV deformity in a case study [10].

Reliable and objective biomechanical evaluations play an important role in reaching positive clinical results for patients with musculoskeletal dysfunction such as OA of the knee joint and foot HV deformity [11,12]. In addition, various kinesiologic problems that develop in knee joints due to the performance of daily activities or walking can cause other musculoskeletal dysfunctions and decrease the quality of life of patients with HV [13,14]. Previous studies have verified the relationship between HV deformity and the kinetic and kinematic values of knee joints, focusing on the measurement of the angle using an anteroposterior radiographic image [5], lower extremity muscle strength [15], or lower extremity alignment [16]. Previous gait studies on the kinematics of the lower extremity joint in HV patients have mainly focused on the foot and ankle complex [17,18]. However, studies verifying the effect of HV angle on the biomechanics of the knee joint during walking are limited and very rarely seen. A previous study on the kinetic and kinematic characteristics occurring in the knee joint of HV patients using a three-dimensional (3D) gait analysis system has verified the effect through comparison with a control group rather than through the application of a therapeutic intervention [19].

Interventions for knee OA and HV include physical therapy [3], orthosis [4], surgical treatment [20], and medication [3]. Nonsurgical interventions, such as HV orthoses, are first preferred for correction of the first interphalangeal and MTP joints for HV deformity [21]. However, previous research examining the therapeutic effects of several HV orthoses in patients with HV deformity have only confirmed changes in the joint angle, pain, or walking variables [22,23]. Therefore, the aim of this study was to verify the effects of different HV orthosis conditions on the 3D kinetics and kinematics of knee joints recognized to contribute to knee OA in patients with HV using two force platforms and a 3D gait analysis system during free walking.

## 2. Materials and Methods

### 2.1. Subjects

The study subjects consisted of twenty-four patients (16 females and 8 males) diagnosed with HV deformation. The average age, height, and weight of all of the participants were 41.2 ± 10.1 years, 162.3 ± 8.5 cm, and 60.0 ± 8.8 kg, respectively. All of the subjects were recruited through a local podiatric hospital and had been treated with conservative management. They were diagnosed with HV deformity within the last 2 years. The inclusion criteria were as follows: (1) no history of foot-toe surgical treatment or severe pain in the foot and ankle joints and segments, and (2) a clinically assessed HV angle of more than 15° in the right and left feet evaluated using a plastic goniometer [22]. The mean HV angle was 22.5 ± 6.2° in the right foot and 21.0 ± 8.3° in the left foot. The minimum and maximum measures of the HV angle among the participants were 18° and 37°, respectively. In addition, the mean values of flexion and extension active range of motion (ROM) of the first MTP joint were 28.3 ± 4.1° and 39.7 ± 6.4°, and the flexion and extension passive ROM values were 42.8 ± 6.0° and 50.2 ± 6.6°, respectively. The subjects voluntarily participated and understood the study purpose and experimental procedures in this study. All of the volunteers were in good health, and there were no problems in conducting the walking experimental process. If the volunteers had severe rheumatoid arthritis, osteoarthritis of the knee joints, or neurological deficits in the knee, foot, and ankle joints and segments, they were excluded from the participation. Those who were taking any medications that might interfere with walking were also excluded. All of the participants completed and submitted written informed consent. The study design and methods were approved by the Institutional Review Board of Jeonju University.

### 2.2. Instrumentation and Data Collection

**Figure 1:**
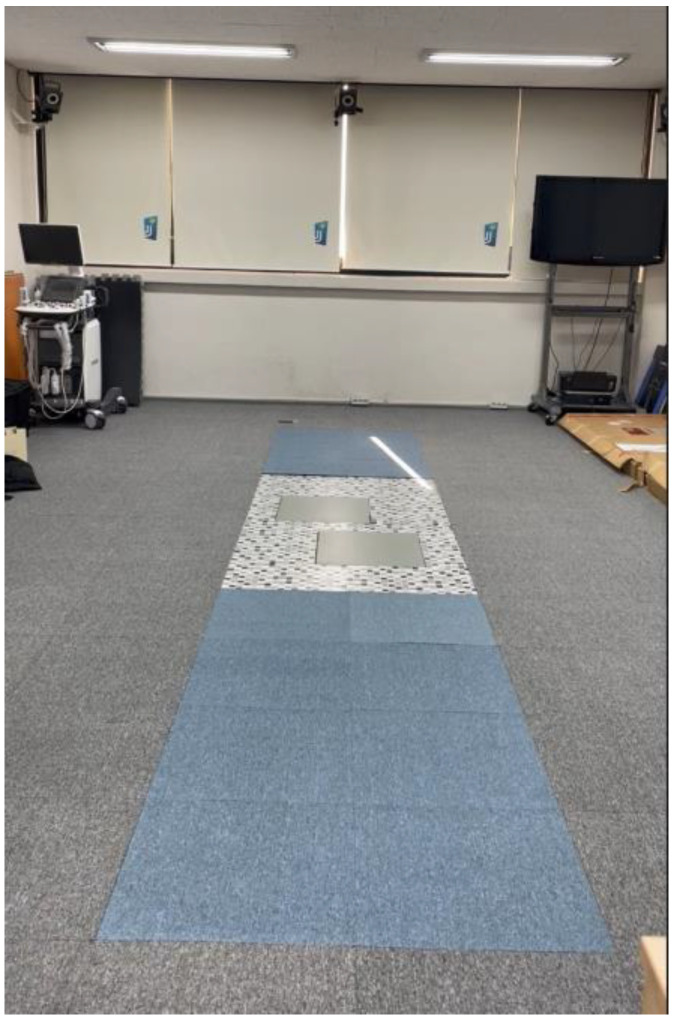
Three-dimensional motion capture cameras with two force platforms embedded in the center of the experimental 6 m walking pathway.

**Figure 2:**
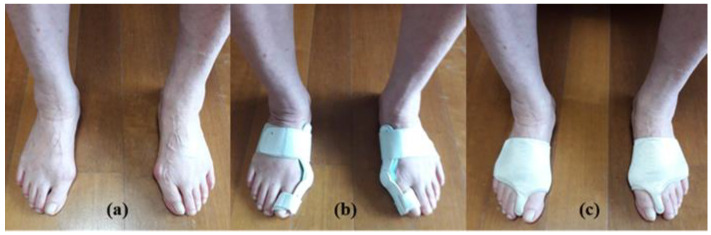
Different foot-toe orthosis conditions: (**a**) without orthosis; (**b**) hard plastic type; (**c**) soft silicone type.

Two force platforms (AMTI, Watertown, MA, USA), established in the middle of the 6 m walking pathway, were used to obtain 3D knee moments (Figure 1). A 3D motion capture system (Vicon Inc., Oxford, UK) that contained eight infrared cameras was operated to collect the kinematic values of both knee joints during walking with and without HV orthoses: without foot-toe orthosis (WTO), with hard plastic orthosis (HPO), and with soft silicone orthosis (SSO). The HPO (Hallufix AG, Grünwald, Germany) consisted of a plastic frame for the medial support of the hallux and first metatarsal bone and a band that straightened the position of the first MTP joint (Figure 2). The SSO (Sanshin Enterprises Co., Tokyo, Japan) was a sock-type orthosis that allowed a soft silicone pad to be placed between the second toe and the hallux (Figure 2). The 3D motion capture camera was set at 100 Hz as the sampling rate.

**Figure 3:**
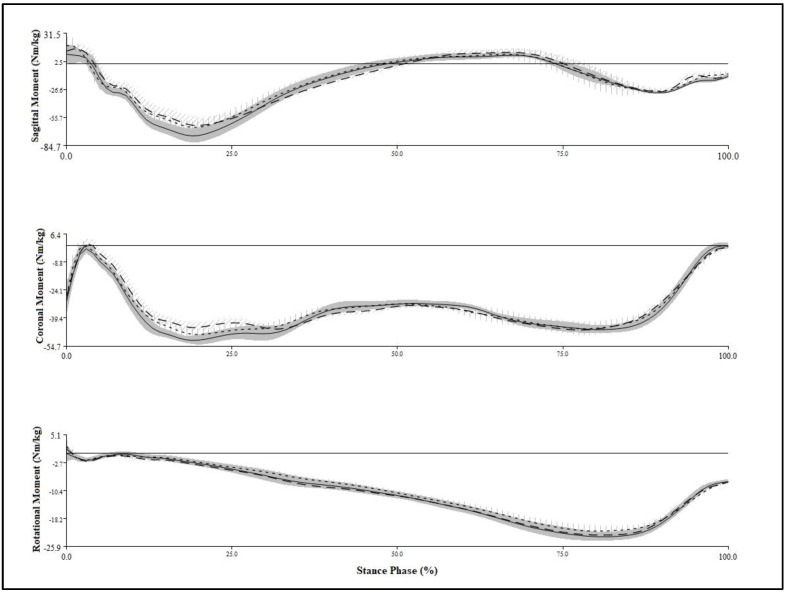
Intra-individual variability in sagittal, coronal, and rotational moments of the knee joint during walking. Average moment from 20 repetitive gait cycles. Without orthosis: solid line with vertical standard deviation band; Hard plastic orthosis: dashed line with diagonal standard deviation band; and Soft-silicone orthosis: dotted line with vertical standard deviation band.

**Figure 4:**
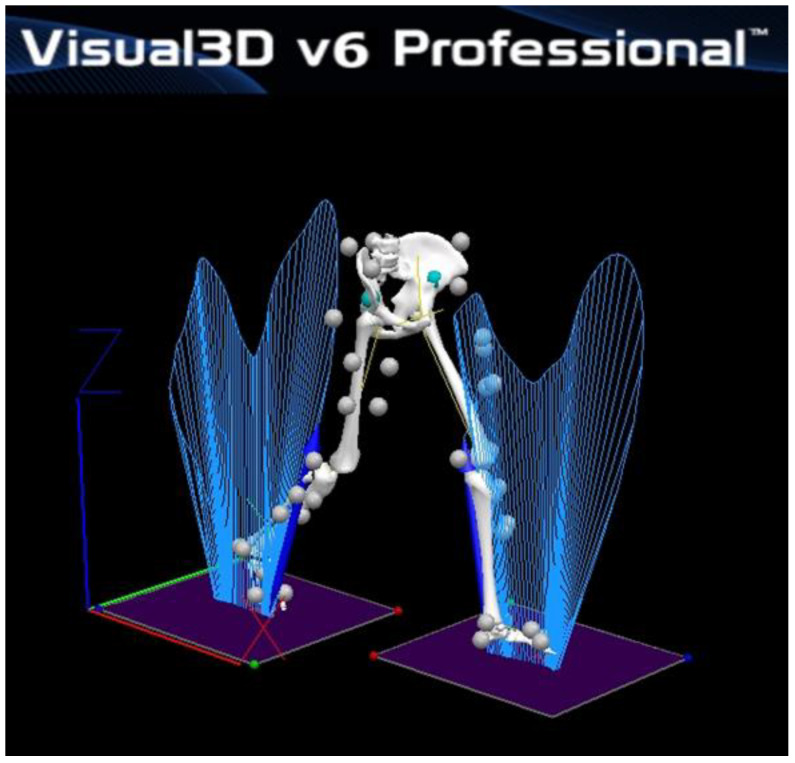
Visual3D representation of reflective markers and 3D ground reaction force butterflies colored blue in laboratory space during walking trials.

A T-figure wand (750 mm) was used for the initial calibration to refer to the object and motion capture system to recognize the 3D origin in the experimental room. Nexus software version 1.8.5 (Vicon Inc., Oxford, UK) was used in the processing of the kinetics and kinematic data obtained from the two force platforms and eight capture cameras. All of the biomechanical data were sent to a central computer for storage and converted to c3d files for use in the final analysis. The c3d data processed in the Nexus program were sent to Visual3D motion analysis software (Visual3D Pro, C-Motion Inc., Germantown, MD, USA) to obtain the final statistical data and graphical reports (Figure 3). The XYZ Cardan sequence was performed to identify the order of the rotations according to the right-hand rule for the segment coordinate system axes [24]. The kinetic and kinematic data of the knee joints were low-pass filtered with a fourth-order Butterworth filter with a cutoff frequency set at 15 Hz and 6 Hz, respectively [24]. The Visual3D software generated virtual lower limb segments and joints of each patient in 3D lab space according to a set of infrared reflective markers that allowed them to calculate the associated knee moments and motions through the entire gait cycle (Figure 4).

### 2.3. Experimental Procedure

**Figure 5:**
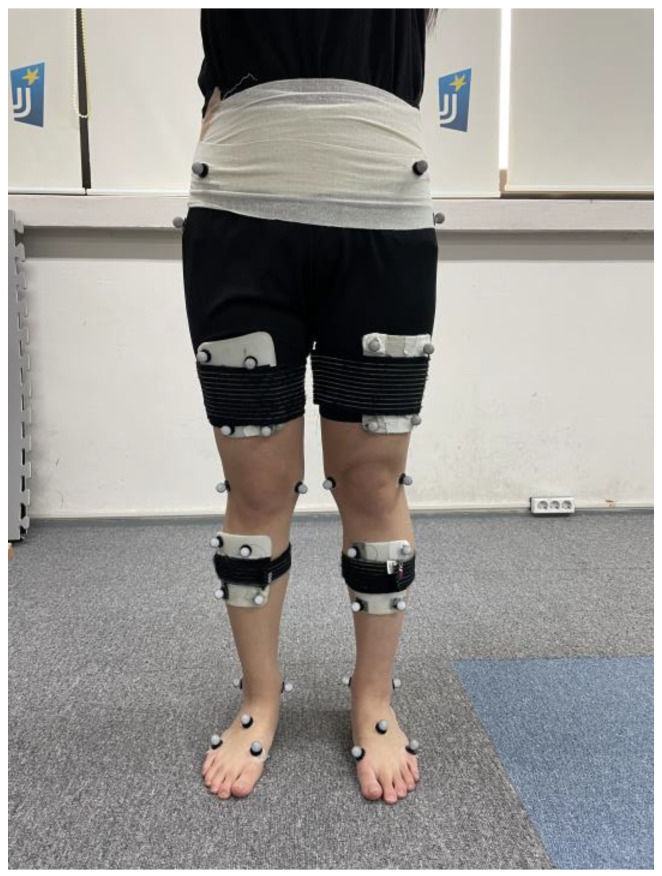
Forty reflective markers were attached to the pelvis and both lower extremities according to the anatomical landmark.

To acquire kinetic and kinematic data through gait analysis, 40 retroreflective markers (1.4 cm) were attached to the right and left lower limbs to greater trochanters, anterior iliac spine, posterior iliac spines, forefeet, midfoot, rearfoot, thigh condyles, and malleoli (Figure 5). Cluster markers composed of 4 reflective markers were fixed to both the calf and thigh segments according to the six degrees of freedom (6DOF) model [24]. Initial static calibration was performed to collect biomechanical data from the knee joints and segments. It was used to make a template imitation to analyze the moment and movement of the knee joints during each gait trial. All of the participants were asked to walk at their own walking speed along an experimental 60 cm walkway set-up in the center of a laboratory room after completing the 3D motion capture system set-up for motion analysis. A total of 8 to 10 walking attempts were performed under each HV orthosis condition until the walking trials were successfully completed. The average kinetic and kinematic values of the knee joints collected from the total gait cycles of the walking trials were used for the final analysis. The successful completion of the walking trial was defined as when each lower extremity accurately stepped on each force platform at the individual’s own walking speed. The calibrated anatomical systems technique was applied to determine the kinematic and kinematic changes that developed in the patient’s knee joint during free walking under three different HV brace conditions. The subjects did not wear shoes or socks other than HV orthosis during the experiment. The order of the application of each HV orthosis was randomly assigned through dice toss.

### 2.4. Statistical Analysis

For the normal distribution test, the Kolmogorov–Smirnov Test was used, and all of the kinetic and kinematic values were normally distributed. Power analysis was performed to determine an appropriate sample size. This sample size was obtained based on an estimated effect size referred to from the previous study [4], which verified the effects of foot orthoses on the kinetic and kinematic variables. A sample size of 25 was determined to be sufficient to identify significance. Repeated-measures ANOVA with Bonferroni’s adjustment was used to compare the kinetic and kinematic values of the knee joint of each HV orthosis condition and both limbs. Post hoc tests were used to determine the pairwise comparison if the ANOVA test results showed the main effect (HV orthosis condition or knee side). The significance level α value was set to 0.05. All of the analyses were executed using IBM SPSS statistics 26.0. Data analysis and collection were conducted by the principal researcher.

## 3. Results

Mauchly’s sphericity test was used to confirm the sphericity assumption, and all of the biomechanical data of the knee joints satisfied the assumption. The mean walking velocities in WTO, HPO, and SSO conditions for all patients were 1.29 ± 0.13 m/s, 1.29 ± 0.13 m/s, and 1.28 ± 0.12 m/s, respectively. There was no significant difference in step width, step length, or gait speed among the different orthosis conditions (all *p* > 0.05) (Table 1).

### 3.1. Three-Dimensional Kinetic Results of the Knee Joint

Several moment values generated at the knee joint showed significant differences between HV orthosis interventions during walking (*p* < 0.05) (Table 2). The first peak of flexion (F_2,40_ = 5.909, *p* = 0.006) and the first peak adduction moment (F_2,40_ = 6.570, *p* = 0.004) that developed at the initial 0–25% stance during walking were significantly different according to the HV orthosis condition (Table 2). Additionally, there were significant differences in the first peak flexor (*p* < 0.05, 95% CI = 0.02 to 0.08) and first peak adduction moment (*p* < 0.05, 95% CI = 0.03 to 0.12) of the knee joint between WTO and HPO conditions, as seen in the results of the post hoc test (Table 3). However, most of the knee moment variables had no significant differences in terms of the orthosis conditions (*p* > 0.05) (Table 3). There were no interactive effects between knee sides and HV orthosis conditions for any of the knee moment variables (*p* > 0.05) (Table 2).

### 3.2. Three-Dimensional Kinematic Results of the Knee Joint

There was only a significant difference in the maximal external rotation motion of the knee joint according to HV orthosis conditions during walking (*p* < 0.05) (Table 4). The mean and standard deviation of the knee motion variable for each foot-toe orthosis condition are shown in Table 5. There was a significant difference in maximal external rotation motion (*p* = 0.021, 95% CI = −0.94 to −0.31) generated at the knee joint between WTO and HPO interventions during gait (Table 5). However, there were no significant differences in other range of motion values between WTO and SSO interventions in the knee joints (*p* > 0.05) (Table 5). The interaction effects between HV orthosis interventions and both knee sides showed no significant differences in any knee motion variables (*p* > 0.05) (Table 4).

## 4. Discussion

It is necessary to understand the kinetic and kinematic specificity that develop in the musculoskeletal components of the lower limbs during walking for patients with HV [25,26]. The current study evaluated the kinetic and kinematic characteristics of the knee joint during walking through force platforms and a 3D motion capture system, commonly selected as the most reliable evaluation method in various fields of study [12,19,22]. This study has the advantage of performing a comprehensive assessment of knee biomechanics using various types of HV orthoses.

The results showed that HV deformity had affected knee moment and range of motion during gait under the HPO intervention compared to the WTO. Greater changes in the first peak adduction and flexion moment of the knee joint occurred at the beginning of the stance phase under the HPO condition compared to those under the WTO condition. Although the results of the study cannot be directly compared to the results of preceding studies, one previous study has investigated kinetic and kinematic outcomes of the lower extremity segments and joints in patients with HV compared to age- and sex-matched healthy controls using a 3D motion capture system during walking [19] and reported that the HV group has significantly greater mean knee abductor moments at contralateral push off and contralateral heel contact in stance phase during gait than the control group [19], indicating that the presence of HV mainly affects the moment of coronal plane of the knee joint during gait. In the current study, it was also found that the application of HPO can significantly reduce KAMs occurring in the knee joint compared to that before the application of the orthosis, supporting the results of the previous study with a control group. HV is known to be associated with pes planus [27,28,29]. Malalignment and deformation of the toe and foot joints, such as HV and pes planus, may contribute to the development of subsequent malalignment and degenerative arthropathy of the proximal hip and knee joints of the lower extremity [7,8,9,10,29]. In a kinesiological aspect, malalignment of the lower limb joints and segments causes a significant abnormal weight loading distribution on the knee [30]. In the genu varus deformity, the load-bearing line moves further to the medial side from the knee axis, creating an increased KAM. Increased peak KAM can affect the progression of OA on the medial tibiofemoral compartment during standing or walking [12,30]. Therefore, HV deformity has a negative effect on the alignment of knee joints and the development of knee OA. The application of HPO for HV correction reduced KAMs compared to SSO or WTO conditions, indicating that it could have a beneficial effect on the management and prevention of medial knee OA. The knee first peak flexion moment, which occurred in initial heel contact of stance phases, showed a significant increase in the HPO intervention compared to that in other foot-toe orthosis conditions. Although no previous findings have compared our outcomes directly, previous research has measured the foot plantar pressure distribution during walking in individuals with HV compared to that in healthy controls using an electric pressure sensor mat [31]. The healthy control group had a significantly higher plantar pressure in the first metatarsal head at the terminal stance than in the HV group [31], suggesting that the application of HPO in this study could elicit more anteroposterior transfer of the ground reaction force, which concluded in more effective anterior propelling power in the final stance stage of the gait cycle.

In the current study, most of the knee kinematic variables, such as the range of motion, were not affected by the application of the HV orthoses, except for the maximum external rotation that occurred in the 75–100% stance phase during walking. The knee maximal external rotation under the HPO condition was reduced compared to that under the WTO or SSO conditions in this study. Decreasing the knee’s external rotation in the terminal stance of the gait cycle can help reduce the toe-out angle and the load at the first metatarsophalangeal joint [19].

This study has several strengths. Most of the existing research has investigated the changes in biomechanics following HV interventions after surgery of the foot and ankle segments [32,33,34]. This study applied two types of HV orthosis to investigate their effects on knee kinetics and kinematics through the use of advanced technology, such as a 3D motion capture system and force platforms. Additionally, this study showed that the application of HPO reduced the adduction moment of the knee joint during walking compared to other conditions. Therefore, the therapeutic intervention of HPO is expected to subsequently prevent degenerative changes in the knee joint and have a positive effect on the management of knee pain that has already occurred in individuals with HV deformity. Abnormal morphological changes at the foot and toe level due to HV deformity seem to be linked to a kinetic chain with an effect on the biomechanics at the level of the knee joint. In a previous study, the risk of development of knee OA increased 6.46 times with a 1% increase in KAM, as seen in the logistic regression analysis [35].

This study also has some limitations. Although all patients had mild to moderate severity of HV deformation, most of the participants had no severe musculoskeletal pain or symptom in their foot or toe, making it difficult to generalize our findings to all patients with HV. The results of the current study only partially supported the hypothesis since HPO did affect the kinetics and kinematics of the knee joint during walking. Future research is needed to verify the correlations between the HV severity and clinical characteristics of the knee joint in a group of HV patients with knee OA using an objective and quantitative evaluation.

## 5. Conclusions

In summary, this study verified the effects of different HV orthosis interventions on the biomechanical characteristics of the knee joints through reliable and objective methods and equipment. The results of this study showed that the foot-toe orthosis in the type of stronger correction of HV deformity significantly lowered the adduction moment of the knee joint compared to a barefoot or soft type of orthosis during gait. Abnormal moments generated in the knee joint during walking can cause degenerative changes and pain in the knee joint. Therefore, the therapeutic intervention of a hard fixed-type orthosis for HV could contribute to better management of abnormal adduction moment of the knee joint as well as static correction of HV deformity.

## Figures and Tables

**Table 1:** Characteristics of the study participants (N = 24).

Characteristics	Mean ± Standard Deviation
Gender	Male: 8, female: 16
Age (years)	41.2 ± 10.1
Height (cm)	162.3 ± 8.5
Weight (kg)	60.0 ± 8.8
Gait speed (m/s)	WTO: 1.29 ± 0.13, HPO: 1.29 ± 0.13, SSO: 1.28 ± 0.12
Step length (m)	WTO: 1.31 ± 0.09, HPO: 1.33 ± 0.10, SSO: 1.32 ± 0.09
Step width (m)	WTO: 0.12 ± 0.03, HPO: 0.11 ± 0.03, SSO: 0.12 ± 0.02

WTO: without foot-toe orthosis; HPO: hard plastic orthosis; SSO: soft silicone orthosis.

**Table 2:** Comparison of 3D knee moment under foot-toe orthosis condition and knee side in stance phase during gait (repeated measures ANOVA, N = 24).

Knee Moment Values (Nm/kg)	Level	F	*p* Value
Flexion moment 1st peak 0–25% stance	Orthosis conditions	5.909	0.006 *
	Both knee sides	0.052	0.822
	Interaction effect	2.401	0.139
Extension moment peak 0–50% stance	Orthosis conditions	0.732	0.488
	Both knee sides	0.236	0.633
	Interaction effect	0.103	0.752
Elexion moment 2nd peak 50–100% stance	Orthosis conditions	0.280	0.757
	Both knee sides	1.274	0.269
	Interaction effect	0.885	0.427
Adduction moment 1st peak 0–25% stance	Orthosis conditions	6.570	0.004 *
	Both knee sides	0.070	0.794
	Interaction effect	0.705	0.412
Adduction moment 25–75% stance	Orthosis conditions	0.525	0.596
	Both knee sides	0.009	0.926
	Interaction effect	0.815	0.378
Adduction moment 2nd peak 50–100% stance	Orthosis conditions	0.484	0.621
	Both knee sides	1.857	0.111
	Interaction effect	1.369	0.257
External rotation moment peak 0–50% stance	Orthosis conditions	0.817	0.450
	Both knee sides	2.333	0.102
	Interaction effect	2.173	0.124
Internal rotation moment peak 50–100% stance	Orthosis conditions	0.518	0.600
	Both knee sides	0.779	0.389
	Interaction effect	1.333	0.281
	Interaction effect	2.753	0.076

* *p* < 0.05.

**Table 3:** Knee moment values under various HV orthosis conditions in stance phase during walking (N = 24).

Knee Moment (Nm/kg)	WTO	HPO	SSO	95% CI
Flexor moment 1st peak 0–25% stance	0.17 ± 0.11 *	0.22 ± 0.12	0.19 ± 0.12	0.02 to 0.08
Extensor moment peak 0–50% stance	−0.73 ± 0.33	−0.73 ± 0.32	−0.71 ± 0.32	−0.63 to 0.81
Flexor moment 2nd peak 50–100% stance	0.13 ± 0.16	0.14 ± 0.14	0.14 ± 0.15	−0.57 to 0.66
Adductor moment 1st peak 0–25% stance	−0.40 ± 0.18 *	−0.34 ± 0.16	−0.39 ± 0.17	0.03 to 0.12
Adductor moment 25–75% stance	−0.21 ± 0.14	−0.21 ± 0.12	−0.20 ± 0.14	−0.83 to 0.70
Adductor moment 2nd peak 50–100% stance	−0.31 ± 0.16	−0.31 ± 0.16	−0.30 ± 0.17	−0.93 to 0.82
External rotator moment peak 0–50% stance	0.04 ± 0.04	0.04 ± 0.05	0.04 ± 0.04	−0.71 to 0.60
Internal rotator moment peak 50–100% stance	−0.17 ± 0.09	−0.17 ± 0.08	−0.16 ± 0.08	−0.99 to 0.83

* Significant difference between WTO and HPO interventions, *p* < 0.05. WTO: without foot-toe orthosis; HPO: hard plastic orthosis; SSO: soft silicone orthosis; CI: confidence interval.

**Table 4:** Comparison of 3D knee movement by HV orthoses and knee side during gait (N = 24).

Knee Motion (°)	Level	F	*p* Value
Maximal extension	Orthosis conditions	1.133	0.332
	Both knee sides	1.117	0.309
	Interaction effect	0.381	0.686
Maximal flexion	Orthosis conditions	0.989	0.382
	Both knee sides	0.997	0.330
	Interaction effect	0.183	0.834
Total range in sagittal plane	Orthosis conditions	2.119	0.133
	Both knee sides	0.000	0.996
	Interaction effect	0.816	0.449
Maximal adduction	Orthosis conditions	1.170	0.321
	Both knee sides	2.944	0.102
	Interaction effect	0.595	0.557
Maximal abduction	Orthosis conditions	0.318	0.792
	Both knee sides	1.270	0.273
	Interaction effect	0.726	0.490
Total range in frontal plane	Orthosis conditions	0.761	0.474
	Both knee sides	0.012	0.913
	Interaction effect	1.088	0.347
Maximal internal rotation	Orthosis conditions	0.518	0.600
	Both knee sides	2.888	0.122
	Interaction effect	1.782	0.181
Maximal external rotation	Orthosis conditions	6.791	0.003 *
	Both knee sides	2.008	0.147
	Interaction effect	2.080	0.138
Total range in transverse plane	Orthosis conditions	2.356	0.108
	Both knee sides	0.509	0.484
	Interaction effect	2.753	0.076

* *p* < 0.05.

**Table 5:** Knee motion variables according to different orthosis interventions during gait (N = 24).

Knee Motion (°)	WTO	HPO	SSO	95% CI
Maximal extension	3.63 ± 2.59	3.77 ± 2.50	4.11 ± 3.06	−0.33 to 1.41
Maximal flexion	59.68 ± 6.78	60.24 ± 7.18	60.29 ± 7.70	−0.49 to 0.97
Total range in sagittal plane	63.31 ± 6.55	64.01 ± 7.07	64.33 ± 7.19	−0.57 to 0.66
Maximal adduction	2.46 ± 1.49	2.62 ± 1.41	2.53 ± 1.55	−0.19 to 0.77
Maximal abduction	5.14 ± 2.99	5.39 ± 3.65	5.32 ± 2.67	−0.53 to 1.20
Total range in frontal plane	7.60 ± 3.03	8.01 ± 3.88	7.86 ± 2.90	−0.85 to 1.04
Maximal internal rotation	16.91 ± 6.07	16.58 ± 5.95	16.88 ± 6.35	−1.00 to 0.99
Maximal external rotation	2.48 ± 2.82 *	1.94 ± 2.46	2.24 ± 3.10	−0.94 to −0.31
Total range in transverse plane	19.39 ± 6.33	18.53 ± 5.96	19.13 ± 6.97	−0.55 to 0.42

* Significant difference between WTO and HPO interventions, *p* < 0.05. WTO: without foot-toe orthosis; HPO: hard plastic orthosis; SSO: soft silicone orthosis; CI: confidence interval.

## Data Availability

Not applicable.
